# Prostaglandin E2 and Transforming Growth Factor-β Play a Critical Role in Suppression of Allergic Airway Inflammation by Adipose-Derived Stem Cells

**DOI:** 10.1371/journal.pone.0131813

**Published:** 2015-07-15

**Authors:** Kyu-Sup Cho, Jung-Hoon Lee, Mi-Kyung Park, Hye-Kyung Park, Hak-Sun Yu, Hwan-Jung Roh

**Affiliations:** 1 Department of Otorhinolaryngology and Biomedical Research Institute, Pusan National University Hospital, Busan, Republic of Korea; 2 Department of Parasitology, Pusan National University School of Medicine, Yangsan, Republic of Korea; 3 Department of Internal Medicine, Pusan National University Hospital, Busan, South Korea; 4 Department of Otorhinolaryngology and Research Institute for Convergence of Biomedical Science and Technology, Pusan National University Yangsan Hospital, Yangsan, Republic of Korea; National Cancer Institute (INCA), BRAZIL

## Abstract

**Background:**

The role of soluble factors in the suppression of allergic airway inflammation by adipose-derived stem cells (ASCs) remains to be elucidated. Moreover, the major soluble factors responsible for the immunomodulatory effects of ASCs in allergic airway diseases have not been well documented. We evaluated the effects of ASCs on allergic inflammation in asthmatic mice treated with a prostaglandin E2 (PGE2) inhibitor or transforming growth factor-β (TGF-β) neutralizing antibodies.

**Methods and Findings:**

Asthmatic mice were injected intraperitoneally with a PGE2 inhibitor or TGF-β neutralizing antibodies at approximately the same time as ASCs injection and were compared with non-treated controls. In asthmatic mice, ASCs significantly reduced airway hyperresponsiveness, the number of total inflammatory cells and eosinophils in the bronchoalveolar lavage fluid (BALF), eosinophilic inflammation, goblet cell hyperplasia, and serum total and allergen-specific IgE and IgG1. ASCs significantly inhibited Th2 cytokines, such as interleukin (IL)-4, IL-5, and IL-13, and enhanced the Th1 cytokine (Interferon-γ) and regulatory cytokines (IL-10 and TGF-β) in the BALF and lung draining lymph nodes (LLNs). ASCs engraftment caused significant increases in the regulatory T cell (Treg) and IL-10^+^ T cell populations in LLNs. However, blocking PGE2 or TGF-β eliminated the immunosuppressive effect of ASCs in allergic airway inflammation.

**Conclusions:**

ASCs are capable of secreting PGE2 and TGF-β, which may play a role in inducing Treg expansion. Furthermore, treatment with a PGE2 inhibitor or TGF-β neutralizing antibodies eliminated the beneficial effect of ASCs treatment in asthmatic mice, suggesting that PGE2 and TGF-β are the major soluble factors responsible for suppressing allergic airway inflammation.

## Introduction

Asthma is a chronic inflammatory airway disease affecting more than 300 million people worldwide [1]. It is characterized by Th2-mediated eosinophilic inflammation, mucus hypersecretion, and airway hyperresponsiveness (AHR) [1,2]. Excessive activation of Th2 cells is thought to play a major role in the initiation and development of the disease [3]. There is mounting evidence that insufficient suppression of regulatory T cells (Tregs) is responsible for the excessive Th2 response in allergic airway disease [4,5].

Mesenchymal stem cells (MSCs) are ubiquitous multipotent cells abundant in adult bone marrow (BM) and adipose tissue [6,7]. In addition to multi-lineage differentiation potential, MSCs derived from adipose tissue (ASCs) and other MSCs have the unique ability to suppress immune responses and modulate inflammation [8]. Several studies have demonstrated that MSCs can ameliorate allergic airway inflammatory diseases, including asthma [9–11] and allergic rhinitis [12–15]. The immunomodulatory effects of MSCs in allergic airway diseases may be mediated by the upregulation of Tregs and increases in several soluble factors such as indoleamine 2, 3-dioxygenase (IDO), prostaglandin E2 (PGE2), transforming growth factor-β (TGF-β), and interleukin (IL)-10 [16–19]. However, the role of these soluble factors in the suppression of allergic airway inflammation by MSCs remains to be elucidated, and the major soluble factors responsible for the immunomodulatory effects of MSCs in allergic airway diseases have not been well documented.

The purpose of this study was to determine whether PGE2 or TGF-β contributes to the immunomodulatory effects of ASCs in asthmatic mice by evaluating the effects of a PGE2 inhibitor or TGF-β-specific neutralizing antibody (Ab) on allergic inflammation.

## Materials and Methods

### Animals

Five-week-old female C57BL/6 mice were purchased from Samtako Co. (Osan, Republic of Korea, http://www.samtako.co.kr) and bred in a specific pathogen free animal facility. The animal study protocol was approved by the Institutional Animal Care and Use Committee of the Pusan National University School of Medicine.

### Isolation and culture of ASCs

Among the MSCs, ASCs were used because of their abundance, relative ease in harvesting and high proliferation potential. Adipose tissue was obtained from the abdominal fat of C57BL/6 mice, washed extensively with equal volumes of phosphate-buffered saline (PBS) and digested with 0.075% collagenase type I (Sigma, St. Louis, MO) at 37°C for 30 min. Enzyme activity was neutralized using α-modified Eagle’s medium (α-MEM) containing 10% fetal bovine serum (FBS) followed by centrifugation at 1,200 × g for 10 min to obtain a pellet. The pellet was filtered through a 100-μm nylon mesh to remove cellular debris and then incubated overnight at 37°C with 5% CO_2_ in control medium (α-MEM, 10% FBS, 100 unit/ml penicillin, 100 μg/ml streptomycin). Following incubation, the plates were washed extensively with PBS to remove residual non-adherent red blood cells. The resulting cell population was maintained at 37°C with 5% CO_2_ in control medium. One week later, once the monolayer of adherent cells had reached confluence, cells were trypsinized (0.05% trypsin-EDTA; Sigma), resuspended in α-MEM containing 10% FBS, and subcultured at the concentration of 2,000 cells/cm^3^. For the experiments, third- or fourth-passage ASCs was used.

Flow cytometric analysis was used to characterize the phenotype of ASCs. At least 50,000 cells (in 100 μl PBS, 0.5% bovine serum albumin (BSA), 2 mmol/l EDTA) were incubated with fluorescein isothiocyanate-labeled monoclonal Abs against mouse stem cell antigen-1 (Sca-1), CD44, CD90, CD45, CD117, and CD11b (BD Biosciences Clontech, Palo Alto, CA) or with the respective isotype control. After washing, labeled cells were analyzed by flow cytometry using a FACSCalibur flow cytometer and Cell Quest Pro software (BD Biosciences, San Diego, CA). The expression percentage of each marker on ASCs was determined by the percentage of positive events, as determined by the isotype-matched negative control.

ACSs were analyzed for their capacity to differentiate into adipogenic, osteogenic, and chondrogenic lineages, as described previously [20]. For adipogenic and osteogenic differentiation, cells were seeded in 6-well plates at a density of 20,000 cells/cm^2^ and treated for 3 weeks with adipogenic and osteogenic medium. Adipogenic and osteogenic differentiation was assessed using oil red O staining, as an indicator of intracellular lipid accumulation, and alizarin red S staining, as an indicator of extracellular matrix calcification, respectively. Chondrogenic differentiation was induced using the micromass culture technique. Briefly, 10 ml of a concentrated ASC suspension (3 × 10^5^ cells/ml) were plated in the center of each well and treated for 3 weeks with chondrogenic medium. Chondrogenesis was confirmed by immunohistochemistry.

### Mouse model of allergic airway inflammation

**Fig 1:**
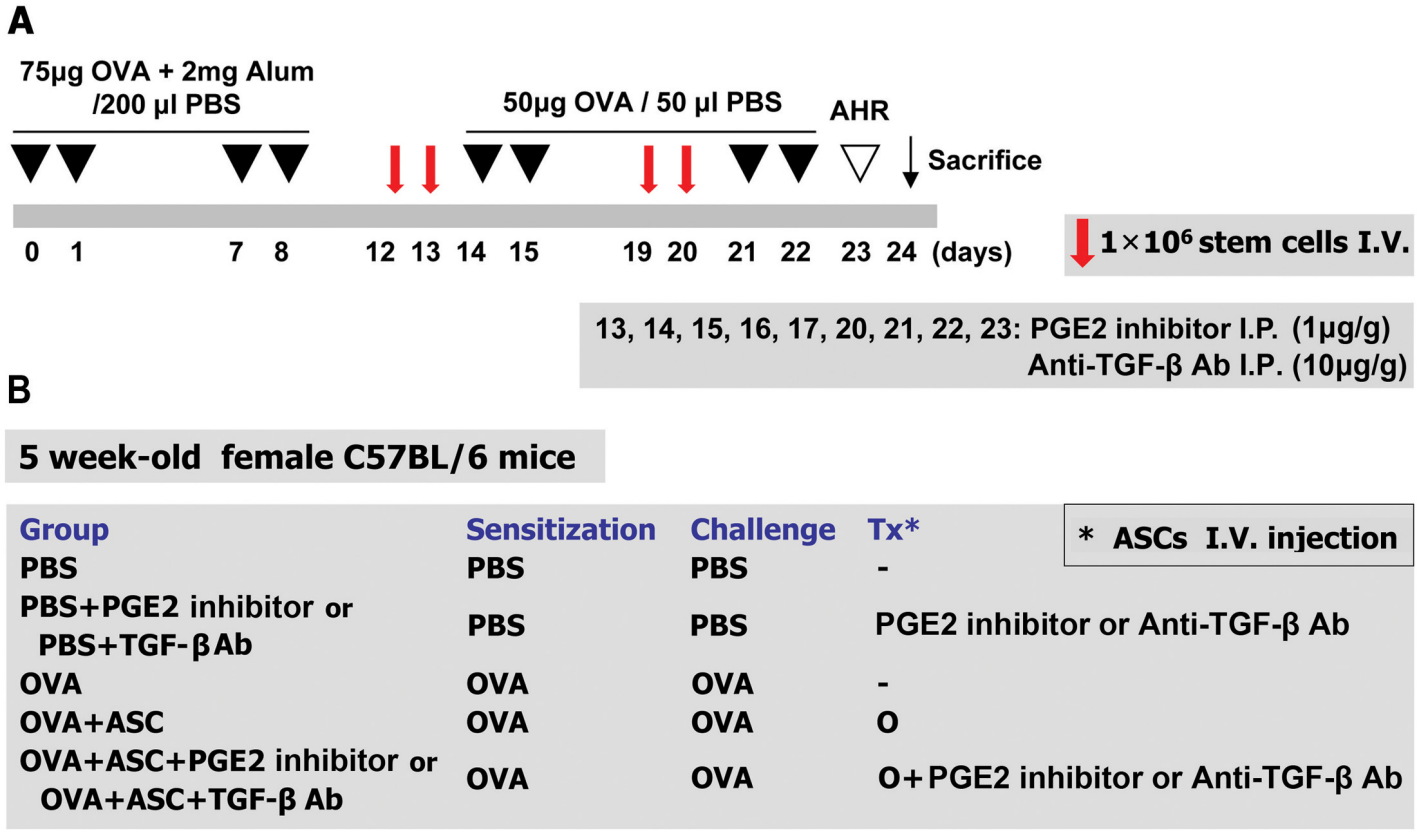
The experimental protocol. (A) Mice were sensitized on days 0, 1, 7, and 8 by intraperitoneal injection of ovalbumin (OVA) and challenged intranasally on days 14, 15, 21, and 22. Purified adipose-derived stem cells (ASCs; 1 × 10^6^) were injected via the tail vein on days 12, 13, 19, and 20. PGE2 and TGF-β were blocked by intraperitoneal injection of a PGE2 inhibitor or anti-TGF-β-Ab on days 13, 14, 15, 16, 17, 20, 21, 22, and 23. (B) The mice were divided into five treatment groups.

A mouse model of allergic airway inflammation was induced as previously reported with minor modification [16,21]. Briefly, mice were sensitized by intraperitoneal injection of 75 μg of ovalbumin (OVA, Sigma, St. Louis, MO, http://www.sigmaaldrich.com) with 2 mg aluminum hydroxide (Sigma) in 200 μl PBS on days 0, 1, 7, and 8. On days 14, 15, 21, and 22 after the initial sensitization, the mice were challenged intranasally with 50 μg OVA in 50 μl PBS (Fig 1A).

### Intravenous transplantation of ASCs and intraperitoneal injection of PGE2 inhibitor and anti-TGF-β Ab

ASCs were washed with PBS and suspended in PBS at a concentration of 1 × 10^7^ cells/ml. To evaluate the effect of ASCs, 0.1 ml purified stem cells were injected with a 26-gauge needle via the tail vein of asthmatic mice once a day on days 12, 13, 19, and 20.

PGE2 or TGF-β were blocked by intraperitoneal injection of a PGE2 inhibitor (1 μg/g body weight in 200 μL PBS) (Cayman Chemical, Ann Arbor, MI) (catalog# 10010088; CAY10526; C_12_H_7_BrO_3_S) or anti-TGF-β Ab (10 μg/g body weight in 200 μL PBS) (R&D Systems, Minneapolis, MN) (catalog# MAB1835; TGF-β1, 2, 3 monoclonal Ab (Clone 1D11), mouse IgG1) on days 13, 14, 15, 16, 17, 20, 21, 22, and 23, respectively (Fig 1A).

Mice were divided into five groups with five mice per group: (a) a PBS group sensitized, pretreated, and challenged with PBS; (b) a PBS+PGE2 inhibitor group or PBS+TGF-β Ab group sensitized and pretreated with PBS followed by treatment with a PGE2 inhibitor or anti-TGF-β Ab, respectively, and then challenged with PBS; (c) an OVA group sensitized with OVA, pretreated with PBS, and then challenged with OVA; (d) an OVA+ASC group sensitized with OVA, pretreated with ASCs, and then challenged with OVA; (e) an OVA+ASC+PGE2 inhibitor group or OVA+ASC+TGF-β Ab group sensitized with OVA, pretreated with ASCs, treated with the PGE2 inhibitor or anti-TGF-β Ab, respectively, and then challenged with OVA. These experiments were repeated four times (Fig 1B).

### Measurement of methacholine AHR

Twenty-four hours after the last challenge, AHR was assessed in conscious, unrestrained mice using non-invasive whole-body plethysmography (Allmedicus, Seoul, Republic of Korea), as described previously [22]. In brief, the mice were placed in the plethysmography chamber and exposed to increasing concentrations of aerosolized methacholine at 0, 12.5, 25 and 50 mg/ml for 10 min. Enhanced pause (Penh) was calculated automatically based on the mean pressure generated in the plethysmography chamber during inspiration and expiration combined with the time of each phase. The Penh values calculated during each 3-min interval were then averaged.

### Differential cell counting in bronchoalveolar lavage fluid

To obtain bronchoalveolar lavage fluid (BALF), the tracheas of anesthetized mice were exposed and cut just below the larynx. A polyurethane flexible tube (0.4 mm in outer diameter, 4 cm in length, and attached to a blunt 24-gauge needle (Boin Medical Co., Seoul, Republic of Korea)) was placed into the trachea, after which the lung was lavaged once with 800 l warm sterile PBS. The BALF samples were centrifuged for 5 min at 1,500 rpm at 4°C. The supernatants were then decanted and frozen immediately at -70°C. Cell pellets were resuspended and washed twice in PBS. The total cell numbers were counted using a hemocytometer. BALF cell smears were prepared using a cytospin apparatus, and stained with Diff-Quik solution (Sysmex Co., Kobe, Japan) to determine the differential cells counts in accordance with conventional morphological criteria. At least 500 cells per slide were evaluated to obtain the differential leukocyte counts.

### Lung histology and inflammation scoring

Lung tissues were removed after the lavage, fixed in 10% neutral formalin for 36 h, and embedded in paraffin. The thin sections of the embedding tissues were stained with hematoxylin and eosin (H&E) and periodic acid-Schiff (PAS) for the identification of eosinophils and for counting mucin-secreting cells, respectively. Lung inflammation was assessed by the degree of peribronchial and perivascular inflammation, which were evaluated on a subjective scale of 0–4, as described previously, [23,24], using the following inflammatory parameters: 0 when no inflammation was detectable; 1 for occasional cuffing with inflammatory cells; 2 when most bronchi or vessels were surrounded by a depth of one to three cells; 3 when most bronchi or vessels were surrounded by a depth of four to five cells; 4 when most bronchi or vessels were surrounded by a depth of more than five cells. For quantifying goblet cell hyperplasia, the percentage of PAS-positive cells in epithelial areas was examined from 8–10 tissue sections per mouse.

### Measurement of serum immunoglobulin

At 48 h after the last OVA challenge, serum was collected from mice via cardiac puncture. Total and OVA-specific immunoglobulins (Ig E, IgG1, IgG2a) were determined by enzyme-linked immunosorbent assay (ELISA) in accordance with the manufacturer’s instructions (R&D Systems, Minneapolis, MN). Absorbance at 450 nm was measured using an ELISA plate reader (Molecular Devices, Sunnyvale, CA).

### Expression of cytokines in the BALF and lung draining lymph nodes

Lung draining lymph nodes (LLNs) were obtained between trachea and both lung lobes. The obtained LLNs were treated with ACK hypotonic lysis buffer (0.15 M NH_4_Cl, 1mM KHCO_3_, 0.1mM Na_2_-EDTA, pH 7.2–7.4) for 2 min at room temperature for lysis of red blood cells (RBCs). After the RBCs were lysed, the remaining cells were filtered using 100-μm mesh (Small Parts Inc., Miramar, FL), and 10^6^ cells/ml were plated in 48 well plates coated with 0.5 μg/ml CD3 Ab (BD Biosciences, San Diego, CA) in RPMI 1640 with 10% fetal bovine serum (FBS) and penicillin/streptomycin. Plated cells were incubated for 72 h at 37°C with 5% CO_2_. After stimulation, the concentrations of mouse IL-4, IL-5, IL-10, IL-13, interferon (IFN)-γ, and TGF-β in the BALF and in the stimulated supernatants of LLNs were examined using commercially available ELISA kits in accordance with the manufacturer's instructions (eBioscience, San Diego, CA). The absorbance of the final reactant was determined at 450 nm using an ELISA plate reader (Molecular Devices).

### Determination of Tregs and intracellular cytokine staining

To evaluate the recruitment of Th1, Th2, and Tregs induced by ASCs treatment, LLN cells from OVA-induced asthmatic mice and ASCs-treated asthmatic mice were cultured in anti-CD3-coated plate for 6 h. To evaluate To evaluate CD4^+^CD25^+^Foxp3^+^ (Tregs) and IL-10^+^/CD4+ T cells, cells were stained with anti-CD4-FITC (0.5 mg/ml) and anti-CD25-APC (0.2 mg/ml) in accordance with the manufacturer’s recommendations (eBiosciences, San Diego, CA). After surface staining, the cells were permeabilized using a Cytofix/Cytoperm Kit (eBiosciences). After permeabilization, the cells were stained with anti-Foxp3-PE-cy7 or anti-IL-10-PE (eBiosciences).

To assess the Th1 and Th2 cell population, LLNs cells were stained with an anti-CD4-FITC Ab. After surface staining, the CD4+ T cells were stained with intracellular anti-IFN-γ-PE-cy7 (eBiosciences) and anti-IL-4-PE (eBiosciences) Abs. Fluorescence was measured using a FACS CantoII cytometer (BD Biosciences) equipped with Canto software (BD Biosciences).

### Statistical analysis

All experiments were repeated a minimum of three times. Data are expressed as mean ± standard error of the mean (SEM). Statistical significance was assessed by the Student’s *t* test or one-way analysis of variance (ANOVA) using the SPSS software package version 18.0 (SPSS Inc., Chicago, IL). A p value <0.05 was considered statistically significant.

## Results

### Characterization of ASC immunophenotype and differentiation

The cultured ASCs from adipose tissue of C57BL/6 mice were negative for the cell surface markers CD45, CD117, and CD11b but positive for Sca-1, CD44, and CD90. These putative ASCs had a spindle shaped fibroblast-like appearance, similar to previously reported adipose tissue and bone marrow-derived MSCs. ASCs had the ability to differentiate into adipogenic, osteogenic, and chondrogenic lineages after culture in the appropriate conditions (data not shown)

### AHR and inflammatory cells in BALF

**Fig 2:**
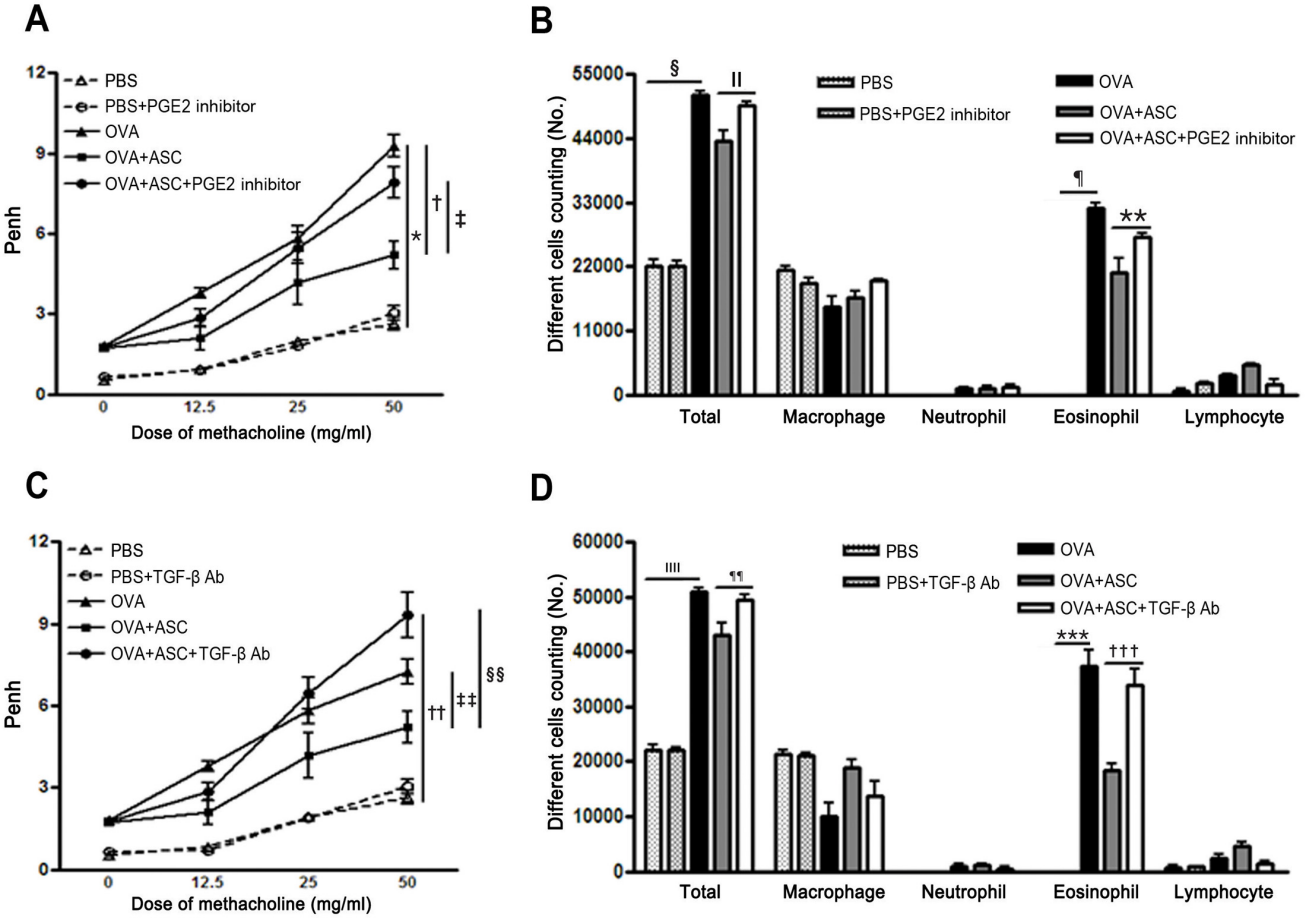
Effect of adipose-derived stem cells (ASCs) on airway hyperresponsiveness (AHR) and inflammatory cells in the bronchoalveolar lavage fluid (BALF). ASCs significantly decreased AHR and the number of total inflammatory cells and eosinophils in asthmatic mice. Treatment with the PGE2 inhibitor or TGF-β neutralizing Ab eliminated the reduction in AHR (A, C) and total cell and eosinophil counts (B, D) induced by ASC treatment. Data are expressed as the mean ± SEM of four independent experiments each performed in triplicate. *,†,§,¶,††,ǁǁ,***,††† *p*<0.001, ‡ *p =* 0.032, ǁ *p* = 0.045, ** *p* = 0.027, ‡‡ *p* = 0.032, §§ *p* = 0.005, ¶¶ *p* = 0.005.

Penh in asthmatic mice increased with the methacholine concentration, and ASC treatment significantly decreased AHR in asthmatic mice. However, treatment with the PGE2 inhibitor or TGF-β-specific neutralizing Ab significantly increased AHR in the OVA+ASC group (*p* = 0.032 and *p* = 0.005, respectively) (Fig 2A and 2C).

The numbers of total inflammatory cells and eosinophils were significantly increased in the BALF of the OVA group compared with the PBS group. However, ASC treatment significantly decreased the numbers of total inflammatory cells and eosinophils in asthmatic mice. However, inhibition of PGE2 production or neutralization of TGF-β failed to reduce BALF total cell numbers (*p* = 0.045 and *p* = 0.005, respectively) and eosinophil counts (*p* = 0.027 and *p*<0.001, respectively) (Fig 2B and 2D).

### Lung inflammation and goblet cell hyperplasia

There was a greater number of eosinophils in the peribronchial and perivascular areas in asthmatic mice. Goblet cell hyperplasia was demonstrated by an increased number and size of goblet cells following PAS staining within the respiratory epithelium in asthmatic mice. However, no obvious infiltration of inflammatory cells and goblet cell hyperplasia were found in asthmatic mice treated with ASCs. Furthermore, ASC administration caused a significant reduction in the inflammation score and PAS-positive cells in asthmatic mice.

**Fig 3:**
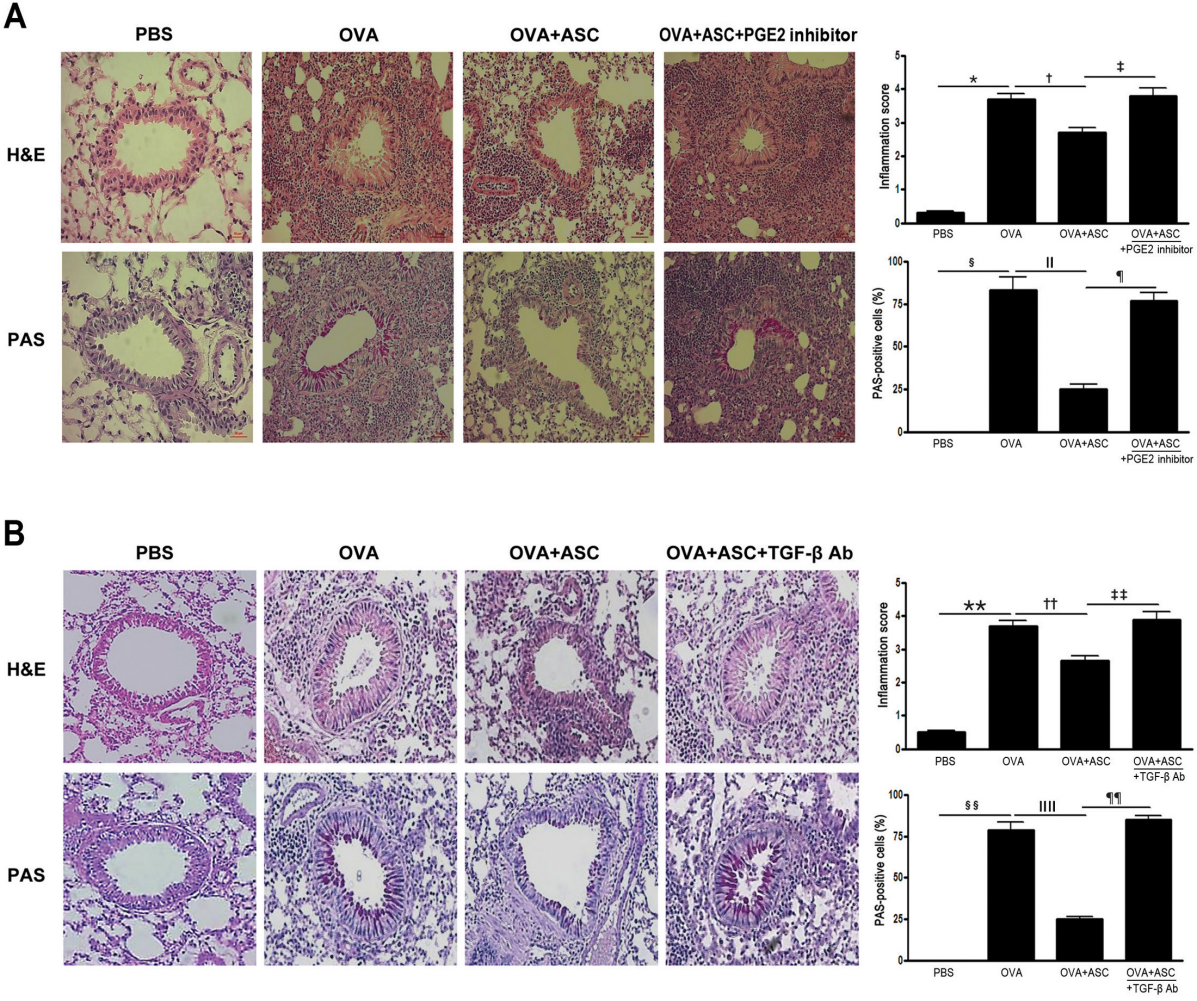
Effects of adipose-derived stem cells (ASCs) on lung inflammation and goblet cell hyperplasia. ASCs treatment decreased the infiltration of eosinophils and PAS-positive cells around the airway and blood vessel in asthmatic mice (H&E, PAS ×200). Blocking PGE2 (A) or TGF-β (B) eliminated the beneficial effect of ASCs on lung inflammation and goblet cell hyperplasia. Data are expressed as the mean ± SEM of four independent experiments each performed in triplicate. *,§,ǁ,**,§§,ǁǁ *p*<0.001, † *p =* 0.020, ‡ *p* = 0.024, ¶ *p* = 0.003, †† *p =* 0.030, ‡‡ *p* = 0.035, ¶¶ *p* = 0.005.

When mice were treated with a PGE2 inhibitor or TGF-β-specific neutralizing Ab, lung inflammation and goblet cell hyperplasia increased in the OVA+ASC+PGE2 inhibitor and OVA+ASC+TGF-β Ab groups compared with the OVA+ASC group. In addition, blocking PGE2 or TGF-β significantly prevented the inflammation score (*p* = 0.024 and *p* = 0.035, respectively) and PAS-positive cell reductions (*p* = 0.003 and *p =* 0.005, respectively) (Fig 3A and 3B) seen in non-treated animals.

### Serum total and OVA-specific IgE, IgG1, and IgG2a

Total and OVA-specific IgE and IgG1 levels were significantly higher in the OVA group than in the PBS group of asthmatic mice. However, systemic administration of ASCs significantly decreased total IgE and OVA-specific IgE in asthmatic mice.

**Fig 4:**
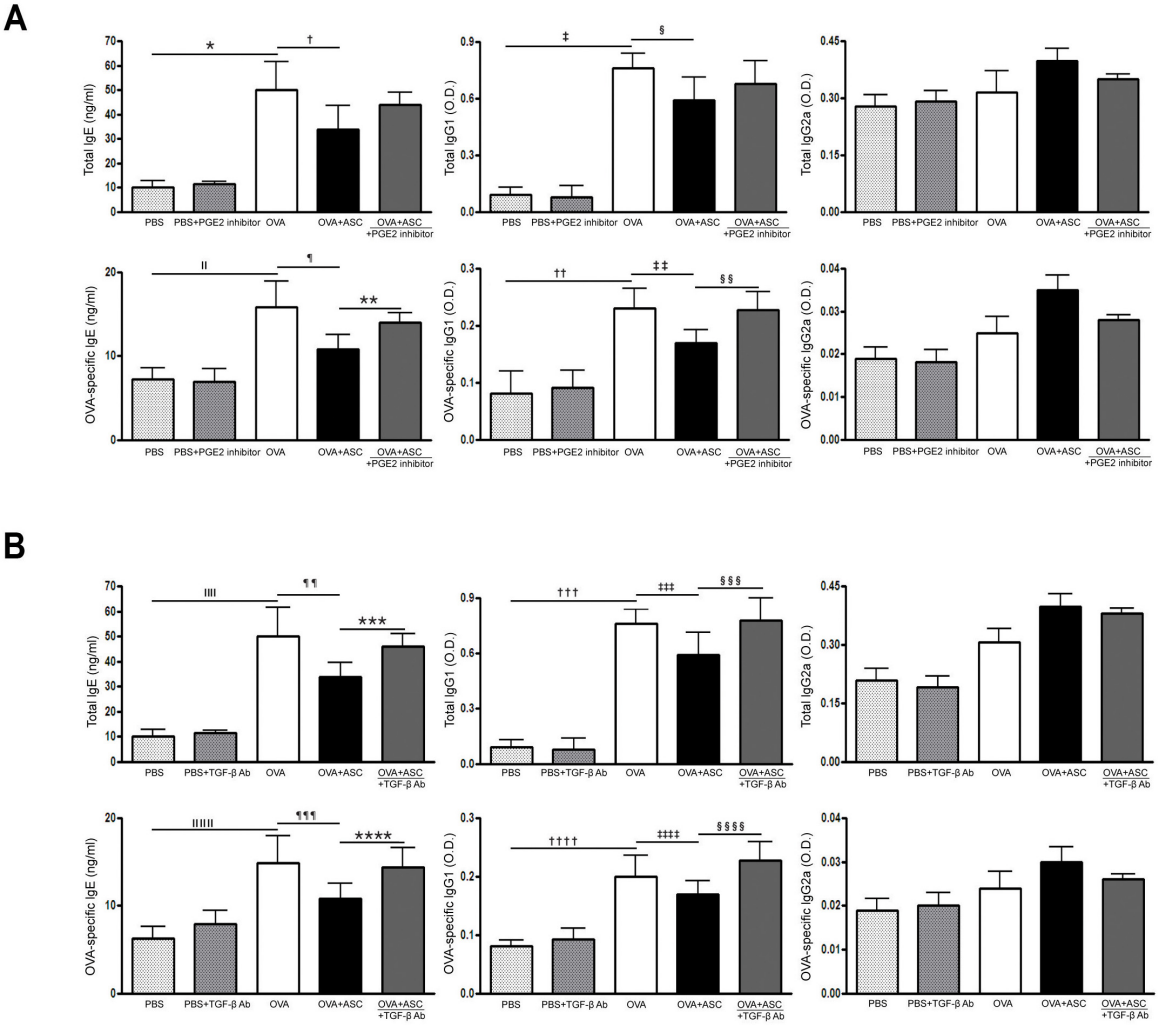
Effect of adipose-derived stem cells (ASCs) on serum levels of immunoglobulin. Systemic administration of ASCs resulted in a significant decrease in total and OVA-specific IgE in asthmatic mice. (A) A PGE2 inhibitor significantly increased OVA-specific IgE and IgG1 in the OVA+ASC group. (B) TGF-β neutralizing Ab resulted in significant increases in total IgE and IgG1 and OVA-specific IgE and IgG1 in the OVA+ASC group. Data are expressed as the mean ± SEM of four independent experiments each performed in triplicate. *,‡,ǁ,††,ǁǁ,†††,ǁǁǁ,†††† *p*<0.001, †,*** *p =* 0.028, § *p =* 0.038, ¶,‡‡,¶¶ *p* = 0.008, **,§§ *p* = 0.009, ‡‡‡ *p* = 0.027, §§§ *p* = 0.037, ¶ *p* = 0.007, **** *p =* 0.035, ‡‡‡‡ *p* = 0.023, §§§§ *p* = 0.032.

The PGE2 inhibitor significantly increased OVA-specific IgE and IgG1 levels in the OVA+ASC group (all *p* = 0.009), but not total IgE and IgG1 levels (Fig 4A). Moreover, neutralization of TGF-β resulted in a significant increase in total IgE and IgG1 (*p* = 0.028 and *p* = 0.037, respectively) and OVA-specific IgE and IgG1 levels (*p* = 0.035 and *p* = 0.032, respectively) in the OVA+ASC group (Fig 4B).

### Cytokine profiles in the BALF and LLN

OVA-challenged mice showed significantly increased levels of IL-4, IL-5, and IL-13 in BALF. However, ASC treatment significantly decreased IL-4, IL-5, and IL-13 in the BALF and LLN of the asthmatic mice. In contrast, ASC treatment significantly increased IFN-γ, IL-10, and TGF-β levels in the BALF and LLN of the asthmatic mice.

**Fig 5:**
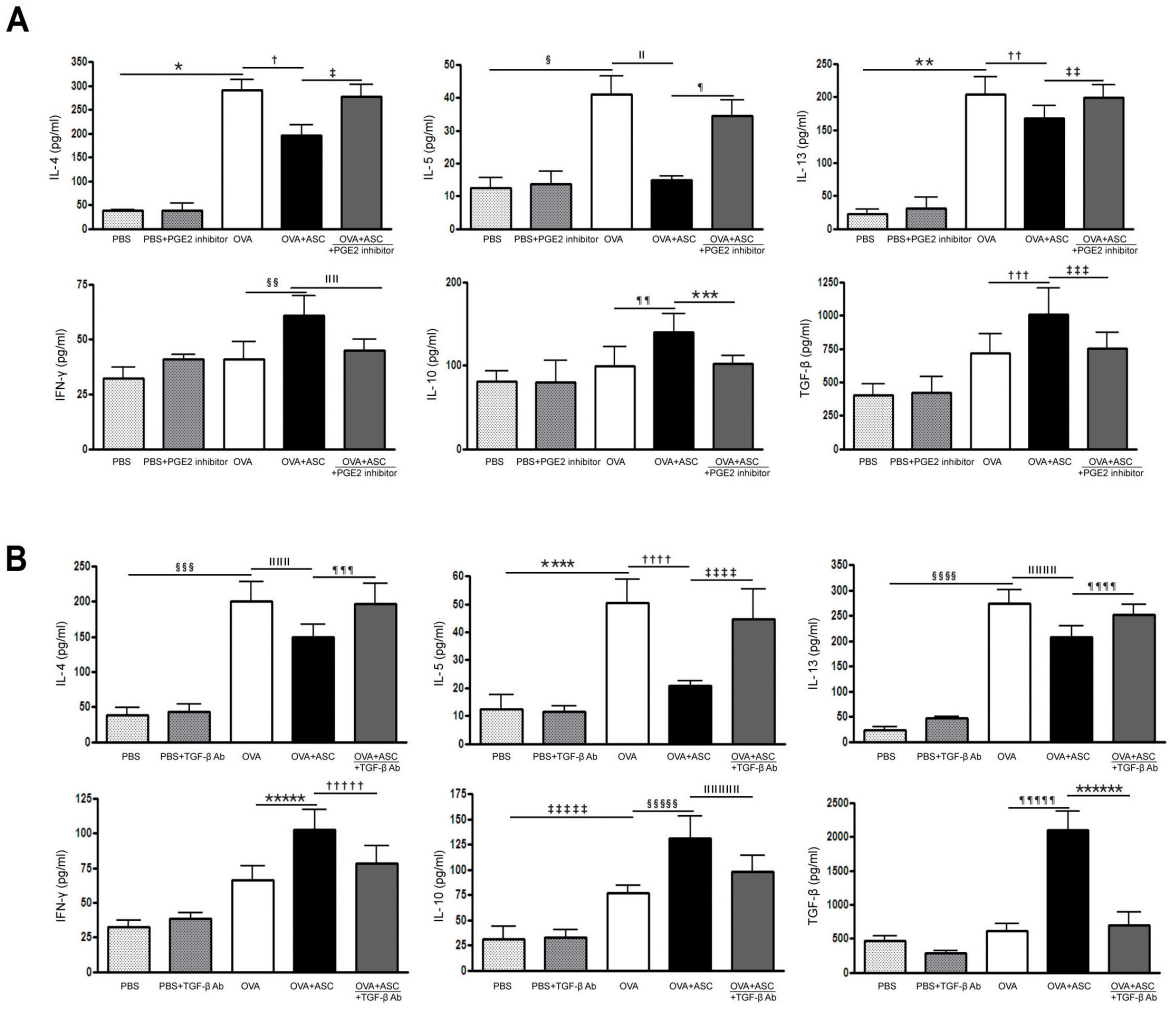
Effect of adipose-derived stem cells (ASCs) on cytokine levels in the bronchoalveolar lavage fluid. IL-4, IL-5, and IL-13 were significantly higher in the OVA group than PBS group. ASC treatment significantly decreased IL-4, IL-5, and IL-13 but increased IL-10 and TGF—β in asthmatic mice. However, the PGE2 inhibitor (A) or TGF-β neutralizing Ab (B) eliminated these immunomodulatory effects of ASCs. Data are expressed as the mean ± SEM of four independent experiments each performed in triplicate. *,§,ǁ,¶,_**,_ §§§,§§§§, ¶¶¶¶¶ *p*<0.001, †,_******_ *p* = 0.007, ‡ *p* = 0.027, ††,¶¶¶ *p* = 0.028, ‡‡ *p =* 0.030, §§ *p =* 0.010, ǁǁ *p* = 0.022, ¶¶ *p* = 0.032, ***,‡‡‡ *p* = 0.038, ††† *p =* 0.049, ǁǁǁ,†††† *p* = 0.003, ****,‡‡‡‡ *p =* 0.004, ǁǁǁǁǁ *p =* 0.026, ¶¶¶¶ *p* = 0.029, ***** *p* = 0.006, ††††† *p* = 0.012, ‡‡‡‡‡ *p* = 0.036, §§§§§ *p =* 0.042, ǁǁǁǁǁ *p =* 0.046.

**Fig 6:**
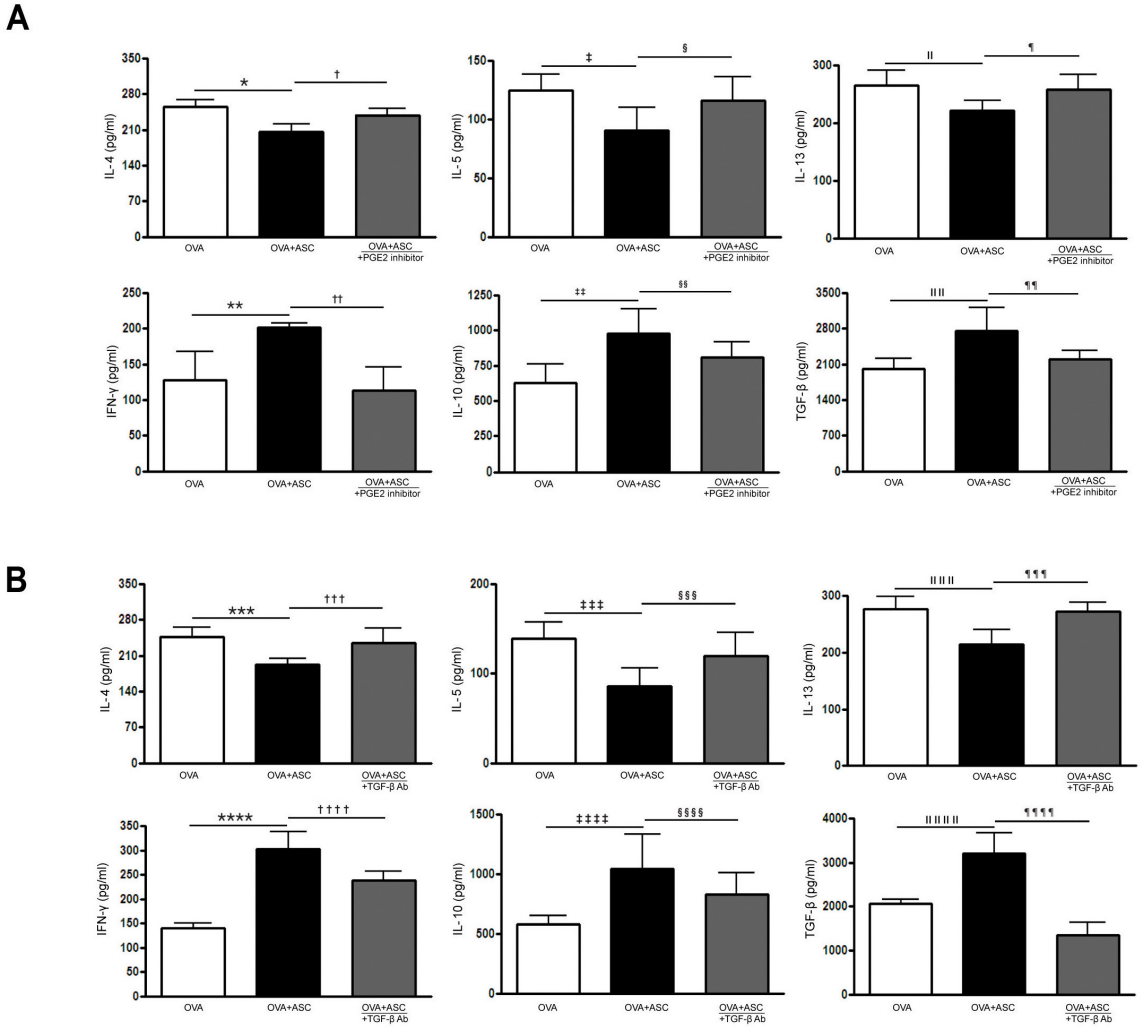
Effect of adipose-derived stem cells (ASCs) on cytokine levels in the lung draining lymph nodes. ASCs treatment significantly decreased IL-4, IL-5, and IL-13 levels but increased IFN-γ, IL-10 and TGF—β levels in asthmatic mice. However, PGE2 inhibitor (A) or TGF-β neutralizing Ab (B) eliminated these immunomodulatory effects of ASCs. Data are expressed as the mean ± SEM of four independent experiments each performed in triplicate. *,*** *p*<0.001, †,†††† *p =* 0.003, ‡,§,ǁǁǁǁ *p =* 0.048, ǁ *p =* 0.037, ¶ *p =* 0.035, **,**** *p =* 0.004, ††,§§§ *p =* 0.009, ‡‡ *p =* 0.032, §§,¶¶ *p* = 0.038, ǁǁ *p =* 0.006, ‡‡‡ *p =* 0.007, ‡‡‡ *p =* 0.002, ǁǁǁ *p =* 0.040, ¶¶¶ *p =* 0.029, ‡‡‡ *p =* 0.049, §§§ *p =* 0.020, ¶¶¶¶ *p =* 0.008.

The PGE2 inhibitor significantly increased IL-4 (*p* = 0.027 and *p* = 0.003, respectively), IL-5 (*p*<0.001 and *p* = 0.048, respectively), and IL-13 (*p* = 0.030 and *p* = 0.035, respectively) levels in the BALF and LLN of the OVA+ASC group. However, IFN-γ (*p* = 0.022 and *p* = 0.009, respectively), IL-10 (*p* = 0.003 and *p* = 0.038, respectively), TGF-β (all *p*<0.0010) levels were significantly decreased after inhibition of PGE2 production in the BALF and LLN of the OVA+ASC group (Figs 5A and 6A). In addition, TGF-β neutralizing Ab significantly increased IL-4 (*p* = 0.028 and *p* = 0.007, respectively), IL-5 (*p =* 0.004 and *p* = 0.009, respectively), and IL-13 (all *p* = 0.029) levels in the BALF and LLN of the OVA+ASC group. However, IFN-γ (*p* = 0.012 and *p* = 0.003, respectively), IL-10 (*p* = 0.046 and *p* = 0.038, respectively), TGF-β (*p =* 0.007 and *p* = 0.008, respectively) were significantly decreased after neutralization of TGF-β in the BALF and LLN of the OVA+ASC group (Figs 5B and 6B).

### T cell populations in the LLN

**Fig 7:**
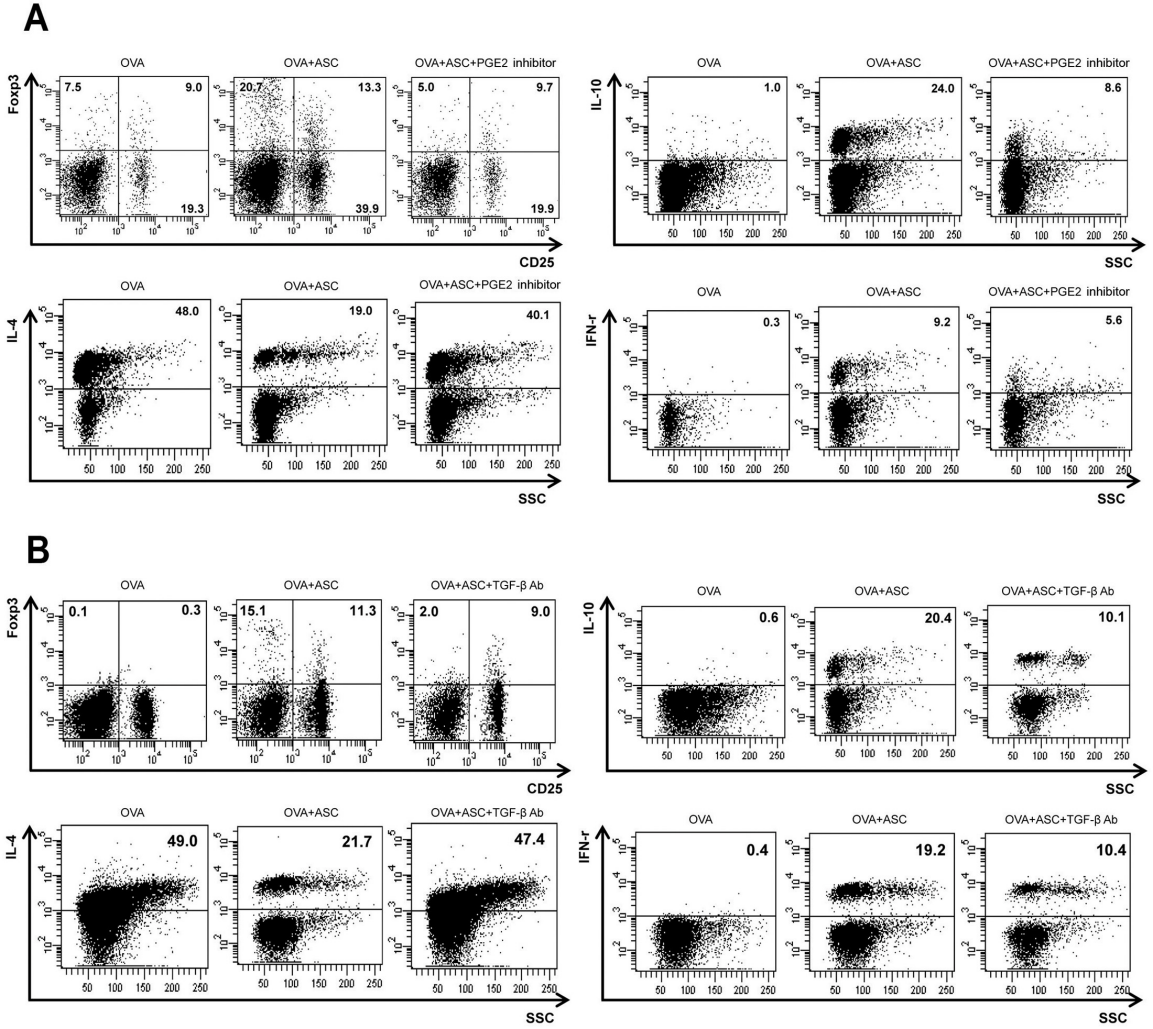
Effects of adipose-derived stem cells (ASCs) on T cells in the lung draining lymph nodes. The CD4^+^ T cells were initially gated and the percentage of IFN-γ^+^, IL-4^+^, IL-10^+^, and CD25^+^ Foxp3^+^ T cells subsequently analyzed. When treating asthmatic mice with PGE2 inhibitor (A) or TGF-β neutralizing Abs (B), blocking of PGE2 and TGF-β prevented the increases in Foxp3^+^CD25^+^, IL-10^+^, and IFN-γ^+^ T cell populations and the decrease in the IL-4^+^ T cell population in the OVA+ASC group.

The populations of CD4^+^CD25^+^Foxp3^+^ T cells and CD4^+^IL-10^+^ T cells were markedly increased by administration of ASCs in asthmatic mice. In the OVA+ASC group, CD4^+^IL-4^+^ T cells were significantly decreased and CD4^+^IFN-γ^+^ T cells were significantly increased compared with the OVA group. Treatment with the PGE2 inhibitor or TGF-β neutralizing Ab prevented the increases in CD4^+^CD25^+^Foxp3^+^, CD4^+^IL-10^+^, and CD4^+^IFN-γ^+^ T cell populations and the decrease in the CD4^+^IL-4^+^ T cell population in the OVA+ASC group (Fig 7A and 7B).

## Discussion

MSCs possess remarkable immunosuppressive properties and can inhibit the proliferation and function of major immune cell populations, including T cells, B cells, and natural killer cells [25,26]. MSCs can also modulate the activities of dendritic cells and induce Tregs *in vivo* and *in vitro* [27,28]. The immunomodulatory function of MSCs has led to increasing interest in using MSCs as promising candidates for the treatment of allergic airway diseases. Several studies have shown that ASC treatment provides a significant reduction in allergic airway inflammation and improved lung function [9–15]. Although the immunomodulatory mechanism of MSCs in allergic airway diseases remains to be elucidated, it has been suggested that the induction and expansion of alveolar macrophages and Tregs play a role in alleviating allergic airway inflammation, while several soluble factors play a role in Tregs differentiation [16–19, 29].

Alveolar macrophages are the predominant immune effector cells at the air-tissue interface in the lungs [30]. Human MSCs cause a pronounced increase in alveolar macrophages, and selective depletion of this macrophage compartment reversed the therapeutic benefit of human MSC treatment on allergic asthma, indicating a critical role for alveolar macrophages in suppressing allergic asthma *in vivo* [29]. Tregs are a unique T cell population with strong immunosuppressive properties, and CD4^+^CD25^+^ Tregs are impaired quantitatively and functionally in allergic airway diseases [31]. CD4^+^CD25^+^ T cells also play an important role in suppressing airway eosinophilic inflammation and in the development of airway hyperreactivity in asthma [17,31]. The induction of Tregs by MSCs involves not only direct contact between MSCs and CD4^+^ T cells, but also the secretion of soluble factors such as IDO, PGE2, and TGF-β [32]. To our knowledge, this is the first study to investigate the potential role of PGE2 and TGF-β in the modulation of the allergic response through the use of antagonist and neutralizing Abs.

The present study demonstrated that intravenous treatment of ASCs in asthmatic mice provides a significant reduction in allergic airway inflammation and an improvement in lung function. The ratio of Tregs in LLNs was higher in the ASC treatment group than in the asthmatic group, which was similar to previous studies that indicated ASCs preferentially activate CD4^+^CD25^+^ T cells subsets, which are the primary mechanisms for the immunosuppressive activity of ASCs [16,17]. Moreover, ASC treatment increased anti-inflammatory cytokines, IL-10 and TGF-β, in the BALF and LLNs, which ultimately led to decreased lung eosinophil infiltration, goblet cell hyperplasia, allergy-specific Th2 cytokines, and Ig production.

Recent studies have suggested a broad role for PGE2 and TGF-β1 in the generation and expansion of Tregs from CD4^+^CD25^-^ precursors [19,32,33]. PGE2 is the cytokine responsible for lymphocyte and Treg expansion [33], while TGF-β is a key regulator of the signaling pathways that initiate and maintain Foxp3 expression and suppressive function in CD4^+^CD25^-^ precursors [34]. Interestingly, human MSCs constitutively express PGE2 and TGF-β1, which are both significantly upregulated by inflammatory mediators [32,35]. Co-culturing T cells with MSCs resulted in elevated levels of PGE2, and treatment with inhibitors of PGE2 production mitigated MSC-mediated immune modulation [36,37]. In this study, a PGE2 synthesis inhibitor abolished the suppressive effect induced by ASCs in asthmatic mice. Together with the secretion of PGE2 by ASCs [37], these findings strongly indicate that PGE2 is involved in the suppression of allergic airway inflammation mediated by ASCs. Nemeth *et al*. noted that neutralizing Abs against TGF-β and BM-MSCs derived from TGF-β1-KO mice eliminated the beneficial effect of BM-MSCs in a mouse model of ragweed-induced asthma, suggesting that BM-MSCs-derived TGF-β1 was responsible for the immunosuppressive effect in asthmatic mice [19]. The present study supported those findings and clarified an important role of TGF-β in the suppression of allergic airway inflammation by ASCs. The present study showed that antagonism of PGE2 production and neutralization of TGF-β eliminated the immunomodulatory effect of ASCs in allergic airway inflammation. When treating mice with a PGE2 synthesis inhibitor or TGF-β-specific neutralizing Abs, treated mice showed no reduction in AHR, inflammatory cells in BALF, lung inflammation, asthma-specific cytokines in the BALF and LLN, or serum Th2 immunoglobulins. Furthermore, blocking of PGE2 and TGF-β resulted in decreased induction of Treg expansion. These results indicate that although the cell contact mechanism might be involved in ASC induction of CD4^+^CD25^+^Foxp3^+^ T cells, soluble factors such as PGE2 and TGF-β play a major role in this process. Furthermore, the present study clarifies the mechanism of ASC-mediated immunosuppression in allergic airway diseases, supporting ASC-derived PGE2 and TGF-β as important molecules involved in the induction of Tregs.

We acknowledge the limitations of our study. For example, although the PGE2 inhibitor or TGF-β-neutralizing Ab may enhance allergic inflammation, we did not include a treatment group sensitized with OVA, treated with PGE2 inhibitor or anti-TGF-β Ab, and then challenged with OVA. To clarify that ASC-derived PGE2 and TGF-β are important mediators in suppression of allergic airway inflammation by ASCs, it will be of value to inject ASCs derived from microsomal PGE2 synthase-1 or TGF-β-deficient mice.

## Conclusions

ASCs are capable of secreting PGE2 and TGF-β, which play a role in inducing Treg expansion. Furthermore, inhibition of PGE2 or TGF-β-specific neutralizing Abs eliminated the beneficial effect of ASC treatment in asthmatic mice, suggesting that PGE2 and TGF-β are the major soluble factors involved in suppressing allergic airway inflammation.
